# Availability and Cost of Naloxone Nasal Spray at Pharmacies in Philadelphia, Pennsylvania, 2017

**DOI:** 10.1001/jamanetworkopen.2019.5388

**Published:** 2019-06-07

**Authors:** Jenny S. Guadamuz, G. Caleb Alexander, Tanya Chaudhri, Rebecca Trotzky-Sirr, Dima M. Qato

**Affiliations:** 1 Institute of Minority Health Research, College of Medicine, University of Illinois at Chicago; 2 Department of Pharmacy Systems, Outcomes and Policy, College of Pharmacy, University of Illinois at Chicago; 3 Department of Epidemiology, Johns Hopkins School of Public Health, Baltimore, Maryland; 4 Center for Drug Safety and Effectiveness, Johns Hopkins School of Public Health, Baltimore, Maryland; 5 Department of Emergency Medicine, Keck School of Medicine, University of Southern California, Los Angeles; 6 Los Angeles County Department of Health Services, Los Angeles, California

## Abstract

**Importance:**

Despite the increasingly important role of pharmacies in the implementation of naloxone access laws, there is limited information on the impact of such laws at the local level.

**Objective:**

To evaluate the availability (with or without a prescription) and cost of naloxone nasal spray at pharmacies in Philadelphia, Pennsylvania, following a statewide standing order enacted in Pennsylvania in August 2015 to allow pharmacies to dispense naloxone without a prescription.

**Design, Setting, and Participants:**

A survey study was conducted by telephone of all pharmacies in Philadelphia between February and August 2017. Pharmacies were geocoded and linked with the American Community Survey (2011-2015) to obtain information on the demographic characteristics of census tracts and the Medical Examiner’s Office of the Philadelphia Department of Public Health to derive information on the number of opioid overdose deaths per 100 000 people for each planning district. Data were analyzed from March 2018 to February 2019.

**Main Outcomes and Measures:**

Availability and out-of-pocket cost of naloxone nasal spray (with or without a prescription) at Philadelphia pharmacies overall and by pharmacy and neighborhood characteristics.

**Results:**

Of 454 eligible pharmacies, 418 were surveyed (92.1% response rate). One in 3 pharmacies (34.2%) had naloxone nasal spray in stock; of these, 61.5% indicated it was available without a prescription. There were significant differences in the availability of naloxone by pharmacy type and neighborhood characteristics. Naloxone was both more likely to be in stock (45.9% vs 27.8%; difference, 18.0%; 95% CI, 8.3%-27.8%; *P* < .001) and available without a prescription (80.6% vs 42.2%; difference, 38.4%; 95% CI, 23.0%-53.8%; *P* < .001) in chain stores than in independent stores. Naloxone was also less likely to be available in planning districts with very elevated rates of opioid overdose death (≥50 per 100 000 people) compared with those with lower rates (31.1% vs 38.5%). The median (interquartile range) out-of-pocket cost among pharmacies offering naloxone without a prescription was $145 ($119-$150); costs were greatest in independent pharmacies and planning districts with elevated rates of opioid overdose death.

**Conclusions and Relevance:**

Despite the implementation of a statewide standing order in Pennsylvania more than 3 years prior to this study, only one-third of Philadelphia pharmacies carried naloxone nasal spray and many also required a physician’s prescription. Efforts to strengthen the implementation of naloxone access laws and better ensure naloxone supply at local pharmacies are warranted, especially in localities with the highest rates of overdose death.

## Key Points

**Question:**

What is the availability of naloxone at pharmacies in Philadelphia, Pennsylvania, 3 years after the implementation of a statewide standing order in Pennsylvania allowing pharmacists to dispense naloxone without a prescription?

**Findings:**

This survey study of 418 pharmacies in Philadelphia found that only one-third carried naloxone nasal spray and many also required a physician’s prescription, including pharmacies in communities with the highest rates of opioid overdose death.

**Meaning:**

Efforts to strengthen the implementation of naloxone access laws and better ensure naloxone supply at local pharmacies are warranted, especially in localities with high rates of opioid overdose death.

## Teaser

This survey study investigates the availability of naloxone nasal spray at pharmacies in Philadelphia, Pennsylvania, 3 years after the implementation of a statewide standing order allowing pharmacies to dispense naloxone without a physician’s prescription.

## Introduction

One of many important strategies to address the opioid epidemic is to increase the distribution of naloxone, a safe and effective reversal agent for opioid overdoses. Within the past 2 decades, every state in the United States has passed a naloxone access law to increase the availability of this life-saving opioid antidote, including a nasal spray form, to laypersons.^1^ However, several reports have documented slow implementation of naloxone access laws in states without a statewide standing order.^2,3^ Pennsylvania, a state with high opioid mortality, was one of the first states to implement a statewide standing order in August 2015, allowing pharmacists to dispense naloxone without a physician’s prescription to any layperson.^4^ Findings from a study conducted between December 2016 and April 2017 in Pennsylvania indicate pharmacist awareness of the statewide standing order for naloxone, however, is lacking, particularly in independent pharmacies.^5^

Despite the increasingly important role of pharmacies in the implementation of naloxone access laws, there is limited information on the impact of such laws, including statewide standing orders, at the local level. We conducted a telephone survey of all pharmacies in Philadelphia, Pennsylvania, between February and August 2017 to examine the availability and out-of-pocket cost of naloxone nasal spray. We were interested in the availability both with and without a prescription from a licensed prescriber, and we examined the association between availability and pharmacy and neighborhood characteristics in Philadelphia. We hypothesized that naloxone was more likely to be available in neighborhoods with elevated rates of opioid overdose death (OOD) and that independent pharmacies would be less likely than retail chains to offer it.

## Methods

### Study Design and Data Source

This study followed the American Association for Public Opinion Research (AAPOR) reporting guideline. A list of all licensed retail pharmacies was obtained from the National Council for Prescription Drug Programs.^6^ In February 2017, we used this list to identify currently active pharmacies and their contact information (via Google searches). We attempted to reach all active pharmacies on this list or a 100% sample of our sampling frame. Criteria for ineligibility included duplicate listing, nonretail (eg, clinic-based) pharmacy or closed-door facility, or pharmacy was permanently closed when we attempted to contact them during our study period. According to the AAPOR guidelines for telephone surveys,^7^ the sampling frame and the eligibility criteria were determined a priori; we provide a flowchart detailing the number pharmacies that were ineligible or did not respond (eFigure in the Supplement). Of 454 eligible pharmacies, 418 participated in our survey (92.1% response rate, using AAPOR response rate 1^7^). We used the American Community Survey (2011-2015) to obtain information about the demographic characteristics of neighborhoods at census tract level and the Medical Examiner’s Office of the Philadelphia Department of Public Health to derive information on the number of OODs per 100 000 people for each planning district.^8,9^ This study was not considered human subjects research by the University of Illinois at Chicago institutional review board.

### Outcomes

Our primary outcome was availability of naloxone nasal spray (hereafter referred to as *naloxone*) with or without an individual prescription. To assess the availability of naloxone, a trained interviewer posing as a customer asked to speak to the pharmacist and asked, “Is naloxone nasal spray or Narcan available or in stock at your pharmacy right now?” If it was not in stock, the interviewer asked whether it could be ordered. If naloxone was in stock, the interviewer then inquired about prescription requirements by asking “Can I get it from your pharmacy without a prescription from my doctor?” If the pharmacy responded affirmatively, the interviewer then inquired about age requirements and the price without insurance (if a range was provided, the lowest price was used).

### Pharmacy and Neighborhood Characteristics

We categorized pharmacies as chains, independents, food stores, or mass retailers based on the National Council for Prescription Drug Programs classification for pharmacy type.^6^ We defined low-income neighborhoods as census tracts in which at least 20% of the population had a household income at or below the federal poverty level or in which the median household income did not exceed 80% of Philadelphia’s median household income ($37 479). We created tertiles for the percentage of population that self-identified as minority (nonwhite). We also derived information on the number of OODs per 100 000 people for each of the 17 residential planning districts in Philadelphia for 2016. Residential planning districts with fewer than 30 OODs per 100 000 population were defined as not elevated (n = 174), districts with 30 to 49 OODs per 100 000 population were defined as elevated (n = 77), and districts with 50 or more OODs per 100 000 were defined as very elevated (n = 167).

### Statistical Analysis

We reported proportions with 95% confidence intervals for naloxone availability and used Pearson χ^2^ tests to compare differences by pharmacy and neighborhood characteristics. We reported medians with interquartile ranges (IQRs) for the cost of naloxone nasal spray. We used a significance value of 5% in all testing; *P* values reported are 2-sided. We conducted the statistical analysis using Stata statistical software version 15 (StataCorp)^10^ and the geospatial analysis using ArcGIS geographic information system version 10.4 (Esri).^11^

## Results

Among the 418 pharmacies surveyed, 60% were located in low-income neighborhoods and 55.0% were independent stores (Table 1). There were no substantial differences between participating and nonparticipating pharmacies by pharmacy and neighborhood characteristics (eTable 1 in the Supplement). One in 3 pharmacies (34.2%) had naloxone nasal spray in stock, with significant differences by pharmacy type and neighborhood characteristics. Naloxone was more likely to be in stock in chain stores (45.9%; 95% CI, 38.2%-53.7%) than in independent stores (27.8%; 95% CI, 22.4%-34.0%). Naloxone was less likely to be available in pharmacies located in predominately minority neighborhoods (28.8%; 95% CI, 21.8%-36.9%) compared with neighborhoods with a large proportion of white residents (40.8%; 95% CI, 33.0%-49.2%). Pharmacies located in planning districts with very elevated rates of OOD were less likely to carry naloxone when compared with those with lower rates of OOD (31.1%; 95% CI, 24.5%-38.6% vs 38.5%; 95% CI, 31.5%-46.0%).

**Table 1.** Availability of Naloxone Nasal Spray at Pharmacies in Philadelphia, Pennsylvania, 2017

Characteristic	Total, No. (%)	Naloxone Nasal Spray Availability, No. (%) [95% CI, %]		
		Available	Not Available	*P* Value^a^
Overall	418 (100)	143 (34.2) [29.8-38.9]	275 (65.8) [61.1-70.2]	
Pharmacy type				
Chain	157 (37.6)	72 (45.9) [38.2-53.7]	85 (54.1) [46.3-61.8]	<.001
Independent	230 (55.0)	64 (27.8) [22.4-34.0]	166 (72.2) [66.0-77.6]	
Food store or mass retailer	31 (7.4)	7 (22.6) [11.0-40.8]	24 (77.4) [59.2-89.0]	
Neighborhood characteristics				
Minority population^b^				
Tertile 1: <41.8%	142 (34.4)	58 (40.8) [33.0-49.2]	84 (59.2) [50.8-67.0]	.10
Tertile 2: 41.8%-89.0%	132 (32.0)	44 (33.3) [25.8-41.9]	88 (66.7) [58.1-74.2]	
Tertile 3: >89.0%	139 (33.6)	40 (28.8) [21.8-36.9]	99 (71.2) [63.1-78.2]	
Not low income^b^^,^^c^	165 (40.0)	60 (36.4) [29.3-44.0]	105 (63.6) [56.0-70.7]	.49
Low income	248 (60.0)	82 (33.1) [27.5-39.2]	166 (66.9) [60.8-72.5]	
Planning district opioid overdose deaths (per 100 000 people)^d^				
Not elevated: <30	174 (41.6)	67 (38.5) [31.5-46.0]	107 (61.5) [54.0-68.5]	.30
Elevated: 30-49	77 (18.4)	24 (31.2) [21.8-42.4]	53 (68.8) [57.6-78.2]	
Very elevated: ≥50	167 (40.0)	52 (31.1) [24.5-38.6]	115 (68.9) [61.4-75.5]	

^a^ Differences tested using χ^2^ tests.

^b^ Five pharmacies had missing neighborhood characteristics.

^c^ Census tracts were defined as low income if at least 20% of the population had household incomes that were below the federal poverty level or if the median household income did not exceed 80% of the median household income in Philadelphia ($37 479).

^d^ Medical Examiner’s Office, Philadelphia Department of Public Health (2016).^8^

Among the 143 pharmacies that stocked naloxone, 61.5% (95% CI, 53.2%-69.2%) indicated it was available without a prescription (Table 2). Naloxone was more likely to be available without a prescription in chain stores (80.6%; 95% CI, 69.6%-88.2%) than in independent stores (42.2%; 95% CI, 30.6%-54.7%). Of the 275 pharmacies that did not have naloxone in stock, 70.5% (95% CI, 64.7%-75.7%) reported they were able to order it. While 91.8% (95% CI, 83.6%-96.1%) of chains reported they could order it, only 60.6% (95% CI, 52.8%-67.9%) of independents were able to do so. Predominately minority and low-income neighborhoods were less likely to offer naloxone without a prescription and less likely to be able to order it when compared with other neighborhoods. For example, only 51.2% (95% CI, 40.4%-62.0%) of pharmacies located in low-income neighborhoods offered naloxone without a prescription, compared with 75.0% (95% CI, 62.3%-84.5%) of pharmacies located in higher-income neighborhoods. Naloxone was less likely to be available without a prescription in neighborhoods that had elevated or very elevated rates of OOD when compared with neighborhoods with lower rates.

**Table 2.** Prescription Requirements and Ability to Order Naloxone Nasal Spray at Pharmacies in Philadelphia, Pennsylvania, 2017

Characteristic	Prescription Required Among Pharmacies Stocking Naloxone Nasal Spray (n = 143), No. (%) [95% CI, %]				Ability to Order Among Pharmacies Not Stocking Naloxone Nasal Spray (n = 268), No. (%) [95% CI, %]^a^			
	Total, No. (%)	Without a Prescription	With a Prescription	*P* Value^b^	Total, No. (%)	Able to Order	Unable to Order	*P* Value^b^
Overall	143 (100.0)	88 (61.5) [53.2-69.2]	55 (38.5) [30.8-46.8]		275 (100.0)	189 (70.5) [64.7-75.7]	79 (29.5) [24.3-35.3]	
Pharmacy type								
Chain	72 (50.3)	58 (80.6) [69.6-88.2]	14 (19.4) [11.8-30.4]	<.001	85 (30.9)	78 (91.8) [83.6-96.1]	7 (8.2) [3.9-16.4]	<.001
Independent	64 (44.8)	27 (42.2) [30.6-54.7]	37 (57.8) [45.3-69.4]		166 (60.4)	97 (60.6) [52.8-67.9]	63 (39.4) [32.1-47.2]	
Food store or mass retailer	7 (4.9)	3 (42.9) [12.8-79.3]	4 (57.1) [20.7-87.2]		24 (8.7)	14 (60.9) [39.7-78.6]	9 (39.1) [21.4-60.3]	
Neighborhood characteristics								
Minority population^c^								
Tertile 1: <41.8%	58 (40.8)	41 (70.7) [57.6-81.1]	17 (29.3) [18.9-42.4]	.15	84 (31.0)	61 (73.5) [62.9-81.9]	22 (26.5) [18.1-37.1]	.20
Tertile 2: 41.8%-89.0%	44 (31.0)	25 (56.8) [41.7-70.7]	19 (43.2) [29.3-58.3]		88 (32.5)	63 (73.3) [62.8-81.6]	23 (26.7) [18.4-37.2]	
Tertile 3: >89.0%	40 (28.2)	21 (52.5) [37.0-67.6]	19 (47.5) [32.4-63.0]		99 (36.5)	61 (64.2) [54.0-73.3]	34 (35.8) [26.7-46.0]	
Not low income^c^^,^^d^	60 (42.3)	45 (75.0) [62.3-84.5]	15 (25.0) [15.5-37.7]	.004	105 (38.7)	78 (75.0) [65.7-82.4]	26 (25.0) [17.6-34.3]	.09
Low income	82 (57.7)	42 (51.2) [40.4-62.0]	40 (48.8) [38.0-59.6]		166 (61.3)	107 (66.9) [59.2-73.8]	53 (33.1) [26.2-40.8]	
Planning district opioid overdose deaths (per 100 000 people)^e^								
Not elevated: <30	67 (46.9)	45 (67.2) [54.9-77.4]	22 (32.8) [22.6-45.1]	.41	107 (38.9)	76 (73.8) [64.4-81.4]	27 (26.2) [18.6-35.6]	.72
Elevated: 30-49	24 (16.8)	13 (54.2) [34.1-73.0]	11 (45.8) [27.0-65.9]		53 (19.3)	37 (69.8) [56.1-80.7]	16 (30.2) [19.3-43.9]	
Very elevated: ≥50	52 (36.4)	30 (57.7) [43.8-70.5]	22 (42.3) [29.5-56.2]		115 (41.8)	76 (67.9) [58.6-75.9]	36 (32.1) [24.1-41.4]	

^a^ Seven pharmacies had missing information.

^b^ Differences tested using χ^2^ tests.

^c^ One pharmacy had missing neighborhood characteristics.

^d^ Census tracts were defined as low income if at least 20% of the population had household incomes that were below the federal poverty level or if the median household income did not exceed 80% of the median household income in Philadelphia ($37 479).

^e^ Medical Examiner’s Office, Philadelphia Department of Public Health (2016).^8^

Similar patterns were observed when we examined the availability of naloxone by neighborhood characteristics for chain and independent pharmacies separately (eTable 2 and eTable 3 in the Supplement). For example, chains located in neighborhoods with very elevated rates of OOD were not only less likely to carry naloxone nasal spray (40.7% vs 46.8%) but were also less likely to offer it without a prescription (77.3% vs 86.1%) when compared with chains located in other neighborhoods.

The median (IQR) out-of-pocket cost for naloxone among pharmacies offering it without a prescription was $145 ($119-$150); costs of naloxone were greatest in independent pharmacies (median [IQR] cost, $147 [$140-$155]) and pharmacies located in planning districts with elevated rates (median [IQR] cost, $150 [$135-$150]) or very elevated rates (median [IQR] cost, $145 [$146-$150]) of overdose deaths (Table 3). In addition, 17.4% (95% CI, 10.7%-27.2%) of pharmacies that did not require a prescription required individuals to be aged 18 years or older to receive it. Such age restrictions were less common in neighborhoods with very elevated OOD rates (13.3%; 95% CI, 4.9%-31.3%).

**Table 3.** Age Restrictions and Out-of-Pocket Cost Among Pharmacies That Offer Naloxone Nasal Spray Without a Prescription

Characteristic	Pharmacies, No.^a^^,^^b^	Out-of-Pocket Cost, Median (IQR), $	*P* Value^c^	Age Restriction, No. (%) [95% CI, %]^d^	*P* Value^e^
Overall	86	145 (110-150)		15 (17.4) [10.7-27.2]	
Pharmacy type					
Chain	56	140 (110-150)	.02	11 (19.6) [11.1-32.4]	.98
Independent	27	147 (140-155)		4 (14.8) [5.5-34.3]	
Food store or mass retailer	3	110 (110-150)		0	
Neighborhood characteristics^f^					
Minority population					
Tertile 1: <41.8%	41	145 (130-145)	.23	9 (22.0) [11.6-37.5]	.24
Tertile 2 41.8%-89.0%	24	145 (110-150)		5 (20.8) [8.7-42.2]	
Tertile 3: >89.0%	20	142 (110-157)		1 (5.0) [0.6-29.9]	
Not low income^g^	44	143 (110-150)	.22	9 (20.5) [10.8-35.3]	.48
Low income	41	145 (130-150)		6 (14.6) [6.6-29.4]	
Planning district overdose mortality rate (per 100 000 people)^h^					
Not elevated: <30	44	140 (110-150)	.30	11 (25.0) [14.2-40.2]	.14
Elevated: 30-49	12	150 (135-150)		0	
Very elevated: ≥50	30	145 (136-150)		4 (13.3) [4.9-31.3]	

Abbreviation: IQR, interquartile range.

^a^ One pharmacy had missing information on cost (N = 87).

^b^ Two pharmacies had missing age restriction data (N = 86).

^c^ *P* values based on statistical significance testing using Mann-Whitney *U* tests.

^d^ Pharmacy restricted access to those aged 18 years or older and required identification before dispensing.

^e^ *P* values based on χ^2^ tests.

^f^ One pharmacy had missing neighborhood characteristics.

^g^ Census tracts were defined as low income if at least 20% of the population had household incomes that were below the federal poverty level or if the median household income did not exceed 80% of the median household income in Philadelphia, Pennsylvania ($37 479).

^h^ Medical Examiner’s Office, Philadelphia Department of Public Health (2016).^8^

The availability of naloxone nasal spray with or without a prescription at Philadelphia pharmacies is depicted in the Figure. Among the 5 planning districts with very elevated rates of OOD, there were notable differences in naloxone availability (eTable 4 in the Supplement). For example, 47.6% of pharmacies in the River Wards area stocked naloxone nasal spray, most of which (90%) offered it without a prescription. In contrast, in the Lower Far Northeast locality, 18.2% of pharmacies stocked naloxone, of which 50.0% offered it without a prescription.

**Figure.**
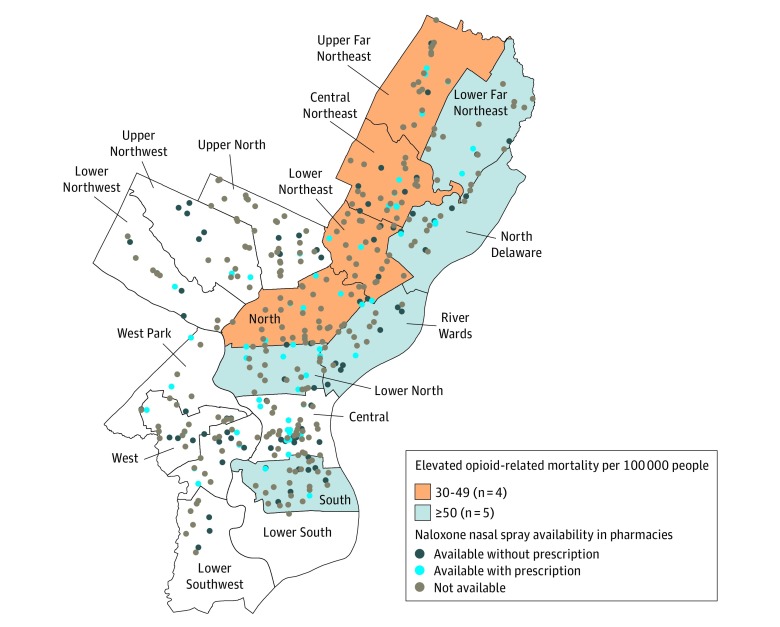
Availability of Naloxone Nasal Spray at Pharmacies in Philadelphia, Pennsylvania, 2017 The map shows the location of Philadelphia pharmacies and availability of naloxone nasal spray (with or without a prescription required). Mortality data are from the Medical Examiner’s Office, Philadelphia Department of Public Health (2016).^8^ Data for naloxone nasal spray availability are from the National Council for Prescription Drug Programs (2015)^6^ and a survey of all pharmacies in Philadelphia performed in 2017.

## Discussion

Despite the widespread implementation of naloxone access laws and important role of naloxone in preventing potentially fatal opioid overdoses, many barriers to its distribution and use remain.^12^ Such barriers are particularly important in communities that have been especially affected by the epidemic and that experience high overdose rates, including in Philadelphia.^13^ We conducted a telephone survey to examine the availability and cost of naloxone nasal spray among all retail pharmacies in Philadelphia in 2017. We found that despite the implementation of a statewide standing order in Pennsylvania more than 3 years prior to our study, only one-third of Philadelphia pharmacies carried naloxone nasal spray and many also required a physician’s prescription. Our findings also suggest that communities with the highest rates of fatal opioid overdose may be less likely to have naloxone access through their local pharmacies.

Although Philadelphia persistently has among the highest rates of OOD in Pennsylvania,^13^ our study indicates that barriers in accessing naloxone at local pharmacies are similar to those identified by Graves et al^5^ in other counties throughout the state. Our findings that independent pharmacies are less likely to stock naloxone and more likely to require a prescription to dispense it are also consistent with statewide estimates.^5^ Although this prior report^5^ did not observe differences across counties within Pennsylvania, our analyses indicate substantial variation in the availability and cost of naloxone across neighborhoods within Philadelphia. Importantly, we found that residents of communities with the highest rates of OOD are more likely to encounter barriers in accessing naloxone at their local pharmacies.

Our findings suggest that naloxone access laws that consist of a statewide standing order are not necessarily more effective than laws that include more restrictive standing order protocols in expanding the availability of naloxone at local pharmacies. For example, when compared with Pennsylvania, more pharmacies in California, a state without a statewide standing order for naloxone, stocked naloxone nasal spray in 2017.^2,5^ In addition, California pharmacies were only slightly less likely to offer naloxone without a prescription than Pennsylvania pharmacies.^2,5^ These findings underscore the importance of increasing pharmacy awareness of statewide standing order protocols and ensuring naloxone is available in their stores.^5^

In response to concerns that pharmacies have failed to facilitate naloxone access, Philadelphia recently introduced a bill that will require all pharmacies to stock naloxone and post a sign notifying the public that naloxone is available in their stores.^14^ Our study provides important baseline information prior to the implementation of this legislation and can be used to guide and target its enforcement, particularly in neighborhoods with the highest rates of fatal opioid overdose that also have disproportionately fewer pharmacies that carry naloxone. While mandating that pharmacies stock naloxone is important, our findings suggest that policies that discourage pharmacies from imposing unnecessary dispensing restrictions, including individual prescription or age requirements, are also critical in these neighborhoods, as are efforts that address the high cost of naloxone. Such efforts may include the distribution of naloxone nasal spray at no cost to laypersons living in low-income neighborhoods with high rates of overdose.^15^

### Limitations

Our analysis has limitations. We only examined the availability of naloxone nasal spray and may therefore underestimate naloxone availability. We focused on the intranasal naloxone formulation because it is widely used and considered the most convenient and easy-to-use option, in particular for laypersons.^16,17^ Its use has increased substantially since it was first approved by the US Food and Drug Administration in November 2015; in 2017 naloxone nasal spray accounted for nearly 70% of all naloxone prescriptions dispensed in the United States.^16^ In addition, the vast majority of naloxone formulations stocked at retail pharmacies in Pennsylvania are for the nasal spray.^5^ State policies and pharmacy practice continue to evolve in response to the epidemic, but pharmacies were surveyed at 1 point in time. Although policies aimed at improving naloxone availability at pharmacies have not changed since the statewide standing order was first implemented in August 2015, since our survey was conducted, Philadelphia has implemented a series of additional programs, including a mass media campaign that started in early 2018, encouraging the public to get and keep naloxone in their homes.^18^

## Conclusions

This study suggests that efforts to strengthen the implementation of statewide standing orders for naloxone and better ensure its supply at local pharmacies are warranted, especially in communities with the highest rates of OOD.

## Notes

Supplement. **eFigure.** Pharmacy Selection **eTable 1.** Characteristics of Pharmacies by Response/Survey Status **eTable 2.** Availability of Naloxone Nasal Spray at Chain Pharmacies in Philadelphia, 2017 **eTable 3.** Availability of Naloxone Nasal Spray at Independent Pharmacies in Philadelphia, 2017 **eTable 4.** Naloxone Availability by Specific Philadelphia Planning Districts, 2017
