# Supplementary material for: Availability and Cost of Naloxone Nasal Spray at Pharmacies in Philadelphia, Pennsylvania, 2017
Source: JAMA Netw Open. 2019 Jun 7;2(6):e195388. doi: 10.1001/jamanetworkopen.2019.5388 (PMC6563583; doi:10.1001/jamanetworkopen.2019.5388)
Supplement: Supplement. — eFigure. Pharmacy Selection eTable 1. Characteristics of Pharmacies by Response/Survey Status eTable 2. Availability of Naloxone Nasal Spray at Chain Pharmacies in Philadelphia, 2017 eTable 3. Availability of Naloxone Nasal Spray at Independent Pharmacies in Philadelphia, 2017 eTable 4. Naloxone Availability by Specific Philadelphia Planning Districts, 2017 [file jamanetwopen-2-e195388-s001.pdf]

Supplementary Online Content

Guadamuz JS, Alexander GC, Chaudhri T, Trotzky-Sirr R, Qato DM. Availability and cost of naloxone nasal spray at pharmacies in Philadelphia, Pennsylvania, 2017. *JAMA Netw Open*. 2019;2(5):e195388. doi:10.1001/jamanetworkopen.2019.5388

eFigure. Pharmacy Selection
eTable 1. Characteristics of Pharmacies by Response/Survey Status
eTable 2. Availability of Naloxone Nasal Spray at Chain Pharmacies in Philadelphia, 2017
eTable 3. Availability of Naloxone Nasal Spray at Independent Pharmacies in Philadelphia, 2017
eTable 4. Naloxone Availability by Specific Philadelphia Planning Districts, 2017

This supplementary material has been provided by the authors to give readers additional information about their work.

© 2019 Guadamuz JS et al. *JAMA Network Open.*

eFigure. Pharmacy Selection

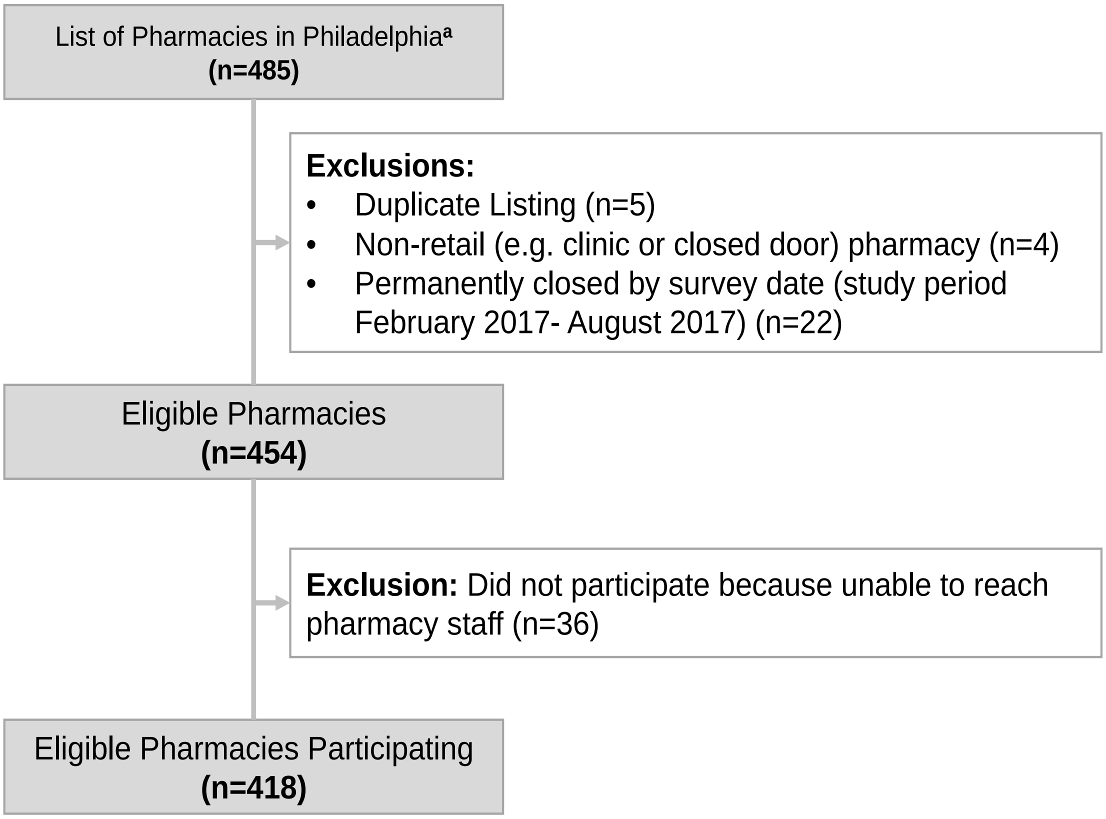

[a] National Council for Prescription Drug Programs, February 2015.[6]

© 2019 Guadamuz JS et al. *JAMA Network Open.*

eTable 1. Characteristics of Pharmacies by Response/ Survey Status

	Pharmacies Surveyed, No. (%)		
	Yes	No	*P*[a]
Overall	418 (92.1)	36 (7.9)	
Pharmacy type			
Chains	157 (37.6)	8 (22.2)	<0.001
Independent	230 (55.0)	22 (61.1)	
Others	31 (7.4)	6 (16.7)	
Neighborhood Characteristics			
Minority Population			
Tertile 1 (<41.8%)	142 (34.4)	6 (17.1)	0.06
Tertile 2 (≥41.8% to <89.0%)	132 (32.0)	17 (48.6)	
Tertile 3 (≥89.0%)	139 (33.7)	12 (34.3)	
Non-low income[b]	165 (40.0)	12 (34.3)	0.51
Low income	248 (60.0)	23 (65.7)	
Planning District Overdose Death Rate[c]			
Not Elevated	174 (41.6)	14 (38.9)	0.65
Elevated	77 (18.4)	5 (13.9)	
Very Elevated	167 (40.0)	17 (47.2)	

Abbreviation: No., number.

[a] Differences tested using Chi-squared tests. [b] Census-tracts are defined as low-income if at least 20% of the population had household incomes that were below the federal poverty level or in which the median household income did not exceed 80% of the median household income in Philadelphia ($37,479.5). [c] Medical Examiner's Office, Philadelphia Department of Public Health (2016).[8]

© 2019 Guadamuz JS et al. *JAMA Network Open.*

eTable 2. Availability of Naloxone Nasal Spray at Chain Pharmacies in Philadelphia, 2017

		Naloxone Nasal Spray Availability, No. (%)							
		Available, In Stock				Not Available, Out-of-stock			
			Prescription required among pharmacies naloxone nasal spray available				Ability to order among pharmacies naloxone nasal spray not available		
	Total, No. (%)	Overall	Without Prescription	With Prescription	*P*[a]	Overall	Can Order	Cannot Order	*P*[a]
Overall	157 (100.0)	72 (45.9)	58 (80.6)	14 (19.4)		85 (54.1)	78 (91.8)	7 (8.2)	
Neighborhood Characteristics									
Minority Population									
Tertile 1 (<41.8%)	63 (40.7)	32 (50.8)	26 (81.3)	6 (18.8)	0.98	31 (49.2)	29 (93.5)	2 (6.5)	0.52
Tertile 2 (≥41.8% to <89.0%)	45 (29.0)	24 (53.3)	19 (79.2)	5 (20.8)		21 (46.7)	20 (95.2)	1 (4.8)	
Tertile 3 (≥89.0%)	47 (30.3)	16 (34.0)	13 (81.3)	3 (18.8)		31 (66.0)	27 (87.1)	4 (12.9)	
Non-low income[b]	75 (48.4)	36 (48.0)	32 (88.9)	4 (11.1)	0.07	39 (52.0)	38 (97.4)	1 (2.6)	0.07
Low income	80 (51.6)	36 (45.0)	26 (72.2)	10 (27.8)		44 (55.0)	38 (86.4)	6 (13.6)	
Planning District Overdose Death Rate[c]									
Not Elevated	77 (49.0)	36 (46.8)	31 (86.1)	5 (13.9)	0.45	41 (53.2)	41 (100.0)	0 (0.0)	0.03
Elevated	26 (16.6)	14 (53.8)	10 (71.4)	4 (28.6)		12 (46.2)	10 (83.3)	2 (16.7)	
Very Elevated	54 (34.4)	22 (40.7)	17 (77.3)	5 (22.7)		32 (59.3)	27 (84.4)	5 (15.6)	

Abbreviation: No., number.

[a] Differences tested using Chi-squared tests. [b] Census-tracts are defined as low-income if at least 20% of the population had household incomes that were below the federal poverty level or in which the median household income did not exceed 80% of the median household income in Philadelphia ($37,479.5). [c] Medical Examiner's Office, Philadelphia Department of Public Health (2016).[8]

© 2019 Guadamuz JS et al. *JAMA Network Open.*

eTable 3. Availability of Naloxone Nasal Spray at Independent Pharmacies in Philadelphia, 2017

	Total, No. (%)	Naloxone Nasal Spray Availability, No. (%)							
		Available, In Stock				Not Available, Out-of-stock			
			Prescription required among pharmacies naloxone nasal spray available				Ability to order among pharmacies naloxone nasal spray not available		
		Overall	Without Prescription	With Prescription	*P*[a]	Overall	Can Order	Cannot Order	*P*[a]
Overall	230 (100.0)	64 (27.8)	27 (42.2)	37 (57.8)		166 (72.2)	97 (58.4)	69 (41.6)	
Neighborhood Characteristics									
Minority Population									
Tertile 1 (<41.8%)	71 (30.9)	25 (35.2)	14 (56.0)	11 (44.0)	0.20	46 (64.8)	28 (60.9)	18 (39.1)	0.21
Tertile 2 (≥41.8% to <89.0%)	74 (32.2)	16 (21.6)	5 (31.3)	11 (68.8)		58 (78.4)	38 (65.5)	20 (34.5)	
Tertile 3 (≥89.0%)	85 (37.0)	23 (27.1)	8 (34.8)	15 (65.2)		62 (72.9)	31 (50.0)	31 (50.0)	
Non-low income[b]	75 (32.6)	22 (29.3)	12 (54.5)	10 (45.5)	0.15	53 (70.7)	33 (62.3)	20 (37.7)	0.209
Low income	155 (67.4)	42 (27.1)	15 (35.7)	27 (64.3)		113 (72.9)	64 (56.6)	49 (43.4)	
Planning District Overdose Death Rate[c]									
Not Elevated	89 (38.7)	30 (33.7)	14 (46.7)	16 (53.3)	0.67	59 (66.3)	31 (52.5)	28 (47.5)	0.493
Elevated	44 (19.1)	7 (15.9)	2 (28.6)	5 (71.4)		37 (84.1)	25 (67.6)	12 (32.4)	
Very Elevated	97 (42.2)	27 (27.8)	11 (40.7)	16 (59.3)		70 (72.2)	41 (58.6)	29 (41.4)	

Abbreviation: No., number.

[a] Differences tested using Chi-squared tests. [b] Census-tracts are defined as low-income if at least 20% of the population had household incomes that were below the federal poverty level or in which the median household income did not exceed 80% of the median household income in Philadelphia ($37,479.5). [c] Medical Examiner's Office, Philadelphia Department of Public Health (2016).[8]

© 2019 Guadamuz JS et al. *JAMA Network Open.*

eTable 4. Naloxone Availability by Specific Philadelphia Planning Districts, 2017

District	Planning District Opioid Overdose Deaths (per 100,000 people)[a]	Total, No. of pharmacies	Naloxone nasal spray availability, No. (%)						Out-of-Pocket Cost Among Pharmacies that Have Naloxone Nasal Spray Available, $	
			Available, In Stock	Prescription required among pharmacies naloxone nasal spray available		Not Available, Out-of-stock	Ability to order among pharmacies naloxone nasal spray not available			
				Without a prescription	With a prescription		Able to order	Unable to order	Mean	Median
River Wards	105.1	21	10 (47.6)	9 (90.0)	1 (10.0)	11 (52.4)	5 (45.5)	6 (54.5)	140	142
North Delaware	75	20	9 (45.0)	6 (66.7)	3 (33.3)	11 (55.0)	4 (36.4)	7 (63.6)	148	145
Lower North	61.5	27	11 (40.7)	3 (27.3)	8 (72.7)	16 (59.3)	8 (50.0)	8 (50.0)	154	150
South	56.7	36	10 (27.8)	8 (80.0)	2 (20.0)	26 (72.2)	8 (30.8)	18 (69.2)	149	145
Lower Far Northeast	53.7	22	4 (18.2)	2 (50.0)	2 (50.0)	18 (81.8)	0 (0.0)	18 (100.0)	131	145
North	44.1	40	8 (20.0)	2 (25.0)	6 (75.0)	32 (80.0)	14 (43.8)	18 (56.3)	150	150
Upper Far Northeast	37.9	22	6 (27.3)	2 (33.3)	4 (66.7)	16 (72.7)	6 (37.5)	10 (62.5)	142	145
Lower Northeast	34.5	27	8 (29.6)	6 (75.0)	2 (25.0)	19 (70.4)	1 (5.3)	18 (94.7)	162	150
Central Northeast	31.7	28	10 (35.7)	5 (50.0)	5 (50.0)	18 (64.3)	9 (50.0)	9 (50.0)	143	145
Lower Northwest	26.7	12	5 (41.7)	4 (80.0)	1 (20.0)	7 (58.3)	2 (28.6)	5 (71.4)	138	145
West	25.9	25	11 (44.0)	9 (81.8)	2 (18.2)	14 (56.0)	3 (21.4)	11 (78.6)	147	150
University Southwest	23.4	19	9 (47.4)	5 (55.6)	4 (44.4)	10 (52.6)	6 (60.0)	4 (40.0)	140	145
Upper Northwest	22.5	18	7 (38.9)	5 (71.4)	2 (28.6)	11 (61.1)	1 (9.1)	10 (90.9)	141	143
Central	19.1	53	23 (43.4)	13 (56.5)	10 (43.5)	30 (56.6)	6 (20.0)	24 (80.0)	148	150
Upper North	18	33	7 (21.2)	6 (85.7)	1 (14.3)	26 (78.8)	10 (38.5)	16 (61.5)	136	147
West Park	15.4	6	3 (50.0)	1 (33.3)	2 (66.7)	3 (50.0)	1 (33.3)	2 (66.7)	141	150
Lower Southwest	15.1	8	2 (25.0)	2 (100.0)	0 (0.0)	6 (75.0)	2 (33.3)	4 (66.7)	166	165
Lower South	Non-residential	1	0 (0.0)	0 (0.0)	0 (0.0)	1 (100.0)	0 (0.0)	1 (100.0)	136	136

Abbreviation: No., number.

[a] Medical Examiner's Office, Philadelphia Department of Public Health (2016).[8]

© 2019 Guadamuz JS et al. *JAMA Network Open.*
